# Impact of [^18^F]FDG-PET and [^18^F]FLT-PET-Parameters in Patients with Suspected Relapse of Irradiated Lung Cancer

**DOI:** 10.3390/diagnostics11020279

**Published:** 2021-02-11

**Authors:** Tine N. Christensen, Seppo W. Langer, Gitte Persson, Klaus Richter Larsen, Annemarie G. Amtoft, Sune H. Keller, Andreas Kjaer, Barbara Malene Fischer

**Affiliations:** 1 Department of Clinical Physiology, Nuclear Medicine & PET, Copenhagen University Hospital, Rigshospitalet, 2100 Copenhagen Ø, Denmark; annemarie.gjelstrup.amtoft@regionh.dk (A.G.A.); sune.hoegild.keller@regionh.dk (S.H.K.); andreas.kjaer@regionh.dk (A.K.); barbara.malene.fischer@regionh.dk (B.M.F.); 2 Cluster for Molecular Imaging, University of Copenhagen, 2200 Copenhagen N, Denmark; 3 Department of Oncology, Copenhagen University Hospital, Rigshospitalet, 2100 Copenhagen Ø, Denmark; Seppo.Langer@regionh.dk; 4 Department of Clinical Medicine, University of Copenhagen, 2100 Copenhagen Ø, Denmark; gitte.persson@regionh.dk; 5 Department of Oncology, Herlev-Gentofte Hospital, University of Copenhagen, 2730 Herlev, Denmark; 6 Department of Pulmonary Medicine, Bispebjerg University Hospital, 2400 Copenhagen NV, Denmark; klaus.richter.larsen@regionh.dk; 7 The PET Centre, School of Biomedical Engineering and Imaging Science, King’s College London, London SE1 7EH, UK

**Keywords:** FDG-PET/CT, FLT-PET/CT, lung cancer, SUV_max_, MTV, PTV, prognosis, relapse diagnosis

## Abstract

Radiation-induced changes may cause a non-malignant high 2-deoxy-2-[^18^F]fluoro-d-glucose (FDG)-uptake. The 3′-deoxy-3′-[^18^F]fluorothymidine (FLT)-PET/CT performs better in the differential diagnosis of inflammatory changes and lung lesions with a higher specificity than FDG-PET/CT. We investigated the association between post-radiotherapy FDG-PET-parameters, FLT-PET-parameters, and outcome. Sixty-one patients suspected for having a relapse after definitive radiotherapy for lung cancer were included. All the patients had FDG-PET/CT and FLT-PET/CT. FDG-PET- and FLT-PET-parameters were collected from within the irradiated high-dose volume (HDV) and from recurrent pulmonary lesions. For associations between PET-parameters and relapse status, respectively, the overall survival was analyzed. Thirty patients had a relapse, of these, 16 patients had a relapse within the HDV. FDG-SUV_max_ and FLT-SUV_max_ were higher in relapsed HDVs compared with non-relapsed HDVs (median FDG-SUV_max_: 12.8 vs. 4.2; *p* < 0.001; median FLT-SUV_max_ 3.9 vs. 2.2; *p* < 0.001). A relapse within HDV had higher FDG-SUV_peak_ (median FDG-SUV_peak_: 7.1 vs. 3.5; *p* = 0.014) and was larger (median metabolic tumor volume (MTV_50%_): 2.5 vs. 0.7; 0.014) than the relapsed lesions outside of HDV. The proliferative tumor volume (PTV_50%_) was prognostic for the overall survival (hazard ratio: 1.07 pr cm^3^ [1.01–1.13]; *p* = 0.014) in the univariate analysis, but not in the multivariate analysis. FDG-SUV_max_ and FLT-SUV_max_ may be helpful tools for differentiating the relapse from radiation-induced changes, however, they should not be used definitively for relapse detection.

## 1. Introduction

The early and precise diagnosis in patients suspected for lung cancer relapse is essential, as a curative intended treatment might be feasible [1]. The 2-deoxy-2-[^18^F]fluoro-d-glucose (FDG)-PET/CT is recommended in patients with a clinical suspicion of recurrence of lung cancer after treatment [2,3]. However, a pathological confirmation is not always feasible. Concerns of risks associated with performing a biopsy and possibilities of sampling errors in small tumors rule out a biopsy in some patients. Therefore, a better accuracy with non-invasive procedures is warranted. Recently, we have shown that 3′-deoxy-3′-[^18^F]fluorothymidine (FLT)-PET/CT adds a diagnostic value in patients who have been treated with definitive radiotherapy [4].

The value of semi-quantitative analysis of FDG-PET/CT after radiotherapy has been sparsely investigated [5,6,7,8]. FDG-SUV_max_ is higher in relapses than in radiation-induced changes [5,6]. However, even a moderate to intense FDG-uptake may be caused by radiation-induced changes rather than recurrence [7], and SUV_max_ ≥ 2.5 has been described 2 years after radiotherapy without evidence of recurrence [8].

During and after radiotherapy, the FLT-uptake decreased more rapidly and more pronounced than the FDG-uptake in several cancers [9,10,11,12], including lung cancer [13,14,15,16]. With a higher specificity and better discrimination between inflammatory lesions and lung cancer [17], the FLT-uptake may reflect a relapse-status better than FDG-uptake. To our knowledge, post-radiotherapy FLT-PET-parameters have only been investigated in two studies: (1) After stereotactic body radiotherapy (SBRT), FLT-SUV_max_ was higher in recurrent lesions than in non-recurrent lesions in a study with 10 patients [18]. (2) After carbon-ion radiotherapy, FLT-SUV_max_ was not significantly different in recurrent and non-recurrent lesions in a study of 19 patients [19].

The prognostic values of FDG-PET-parameters after radiotherapy are not in agreement. Lopez Guerra et al. [20] showed a prognostic value of post-radiotherapy FDG-SUV_max_ for overall survival, and Bollineni et al. [21] showed a trend towards a prognostic value of survival of FDG-SUV_max_ 3 months after SBRT. Other studies did not find any prognostic value of FDG-PET neither in non-small cell lung cancer (NSCLC) [22] nor small cell lung cancer (SCLC) [23].

In this study, we explore the associations between FDG-PET- and FLT-PET-parameters after radiotherapy and patient outcome, to assess if the quantitative analysis can add value to the diagnosis of relapse after radiotherapy and to evaluate the prognostic value of FDG- and FLT-PET-parameters.

## 2. Materials and Methods

### 2.1. Patients

Patients were eligible if they met the following inclusion criteria: Histologically confirmed lung cancer, i.e., NSCLC or SCLC; definitive radiotherapy within 2 years and current suspicion of local relapse based on symptoms and/or CT- or FDG-PET/CT warranting (further) FDG-PET/CT for relapse diagnosis.

Patients were recruited prospectively from three university hospitals in the Capital Region of Denmark during January 2015 to 2019. The follow-up ended in May 2020. The study was approved by the local ethics committee (approval number H-4-2014-060) and by institutional review boards. All the patients signed a written informed consent. We have previously published data from this cohort concerning the diagnostic value of visual analysis of PET scans [4].

### 2.2. Imaging

All the patients went through an FLT-PET/CT as well as an FDG-PET/CT within 4 weeks of each other.

FLT-PET/CT was performed at Rigshospitalet on a Siemens Biograph TruePoint TrueV 40 or 64 PET/CT-scanner. FLT (5 mg/kg, max 350 MBq) was injected 60 ± 10 min prior to scanning, according to the study guidelines. Patients were not subject to any restrictions regarding fasting or resting before FLT-PET/CT. FLT-PET were reconstructed using an ordered subset expectation maximization (OSEM) with point spread function modelling (PSF), three iterations, and 21 subsets with a 2 mm full width half maximum (FWHM) Gaussian post-reconstruction filter.

FDG-PET/CT was performed as a routine clinical investigation, according to the local procedures at the referring hospital. Patients fasted at least 4 h before the injection of FDG and rested approximately 60 min between the injection and scan. Images were reconstructed following clinical guidelines for FDG-PET/CT. Details are available in Supplemental Table S1.

### 2.3. Image Analyses

PET/CT-scans were reviewed and analyzed on a Mirada Medical Ltd. XD 3.6 workstation by TNC, AGA, and BMF. The two latter readers being a board certified radiologist and nuclear medicine physician, respectively with more than 10 years of experience.

**Figure 1:**
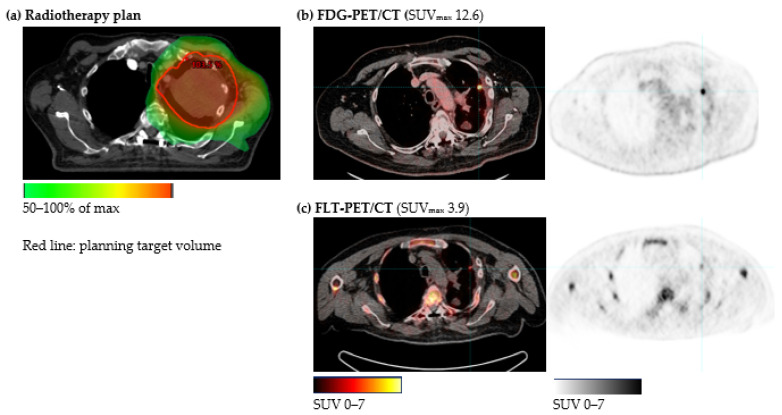
Patient with relapse in the irradiated high-dose volume (HDV). (**a**) HDV defined as the volume irradiated with >50% of max from the radiotherapy plan. The patient received two Gy × 33 for a T3N1M0 tumor in the left upper lobe. FDG-PET/CT (**b**) and FLT-PET/CT (**c**) 7 months after the end of radiotherapy both showed a high tracer uptake. SUV: Standardized uptake value.

**Figure 2:**
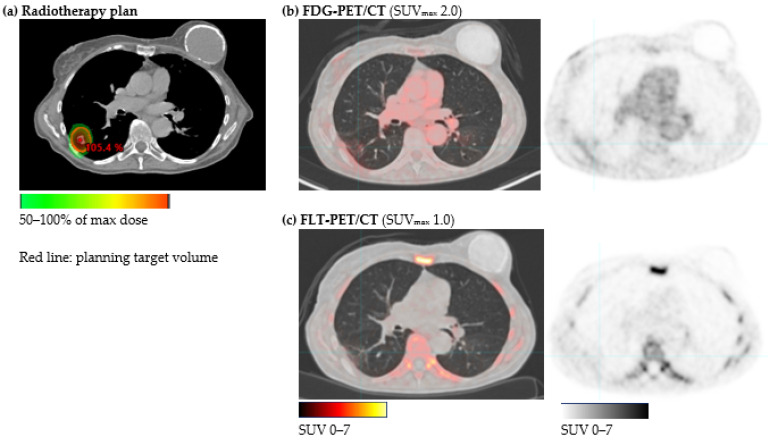
Patient with radiation-induced changes in HDV. (**a**) HDV defined as volume irradiated with >50% of max from the radiotherapy plan. The patient received SBRT (22 Gy × 3) for a T1N0M0 tumor in the right lower lobe. (**b**) FDG-PET/CT and (**c**) FLT-PET/CT 13 months after the end of radiotherapy showed only a faint shadow of FDG- and FLT-uptake in the HDV.

From the radiotherapy-plan, a high-dose irradiated volume (HDV) was defined within the 50% isodose curve (Figure 1 and Figure 2) for each patient. Radiation-induced changes may occur in larger areas than, e.g., the planning target volume, thus to be able to evaluate the potential of FDG-PET/CT and FLT-PET/CT to differentiate radiation-induced changes from relapse, HDV was selected to be more representative of the clinically challenging area. Within HDV, the tracer uptake was quantified by a standardized uptake value (SUV) measuring SUV_max_ and SUV_peak_. SUV_max_ was defined as an SUV in the voxel with the highest SUV in the region of interest. SUV_peak_ was defined as the highest average of SUV in a 1 cm^3^ sphere within the region of interest.

In patients with intrapulmonary relapse, SUV_max_, SUV_peak_, and the functional tumor volume were measured in the relapses. Functional tumor volumes were delineated in three different ways: (1) by 50% of SUV_max_ (MTV_50_ from FDG-PET/CT (metabolic tumor volume) and PTV_50_ from FLT-PET/CT (proliferative tumor volume), as recommended for FDG-PET by the European Association of Nuclear Medicine (EANM) procedure guidelines [24]; (2) by 80% of SUV_max_ (MTV_80_ and PTV_80_) as suggested for post-radiotherapy delineation for better discriminating from the adjacent tissue [25]; and (3) by SUV_max_ > 3.0 (MTV_3.0_ and PTV_3.0_) as this threshold previously has shown promise in the post-treatment setting [26]. In patients with more than one intrapulmonary malignant lesions, the highest SUV_max_ and SUV_peak_ from the lesions were used, and the functional tumor volume was calculated as the sum of all malignant lesions.

### 2.4. Outcome

The endpoints were (1) relapse and (2) overall survival. Relapses were categorized as (1) within HDV, (2) intrapulmonary, and (3) extra-pulmonary. The relapse status was defined retrospectively as the presence or absence of relapse within 6 months after inclusion. The relapse status was preferably confirmed by histology. If histology was not available or inconclusive, the relapse status was judged by a clinical oncologist, based on subsequent imaging procedures, invasive procedures, histology, and conference decisions within 6 months. The clinical oncologist was blinded for the name and age of the patient, names of the involved physicians, and dates of investigations.

The overall survival was calculated from the day of relapse suspicion to death by any cause.

### 2.5. Statistics

The correlation analyses were performed using Spearman’s correlation as the PET-parameters were not normally distributed. Differences of PET-parameters in HDVs with relapse and non-relapsed HDVs, and differences of PET-parameters in relapsed lesions within vs. outside of HDV were estimated with independent t-tests or the Welch test, if assumptions for the t-test were not fulfilled. Receiver operating characteristic (ROC) curves was created to explore the diagnostic accuracy of FDG-SUV_max_ and FLT-SUV_max_. The sensitivity and specificity for relapse diagnosis within HDV with variant SUV_max_ cutoffs were estimated. The diagnostic value of combining FDG-SUV_max_ and FLT-SUV_max_ was explored with different combinations of FDG-SUV_max_- and FLT-SUV_max_-cutoffs.

Overall survival was calculated by the Kaplan-Meier method. The univariate and multivariate survival analysis including clinical parameters and PET-parameters from relapsed lesions and HDV were performed by Cox regression. PET-parameters were included both as continuous variables and dichotomized by the median cut-off. The number of included covariates in the multivariate analysis was restricted due to the limited number of patients. PET-parameters with *p* < 0.150 in the univariate analysis were included in addition to preselected clinical covariates: Sex, age (at time of suspicion), and time from the end of radiotherapy to the suspicion of relapse. The correlation analyses were performed separately on FDG-PET and FLT-PET-parameters to avoid including highly correlated parameters. Statistical analyses were performed in SPSS, version 25.

## 3. Results

A total of 63 patients were enrolled in this study. In two patients, the relapse status after 6 months of follow-up was non-confirmed, and they were excluded from further analysis. Accordingly, 61 patients were included in the final analysis. Patients were included based on the relapse suspicion raised on surveillance CT (*n* = 51) or surveillance FDG-PET/CT (*n* = 9). Four patients had symptoms of relapse when included, of these, one patient was included without prior CT. Fifty-seven patients had NSCLC, two patients SCLC, and two patients mixed NSCLC/SCLC. Patients were included a median of 7 months (interquartile range (IQ): 5–12) after the end of radiotherapy.

FDG-PET/CT was performed a median of 21 days [IQ: 15–27] after relapse was suspected, and FLT-PET/CT was performed a median of 23 days [IQ: 21–29] after relapse was suspected. FDG-PET/CT and FLT-PET/CT were conducted a median of 6 days apart (IQ: 3–9 days). Patient characteristics are presented in Table 1.

Thirty patients were diagnosed with intrapulmonary relapse within 6 months after inclusion. In 16 patients, the relapse was located within the HDV. Figure 1 and Figure 2 illustrate the radiotherapy plan and FDG-PET/CT and FLT-PET/CT from two patients with relapse within HDV and without relapse, respectively. The relapse was confirmed by histology in eight patients (four HDVs) and by subsequent growth in 11 patients (six HDVs). In twelve patients, the relapse diagnosis was based solely on FDG-PET/CT as decided by a multi-disciplinary conference, of these, six patients had the disseminated disease. Two patients were diagnosed with extra-pulmonary relapse only.

### 3.1. FDG-SUV_max_ *and* FLT-SUV_max_

**Figure 3:**
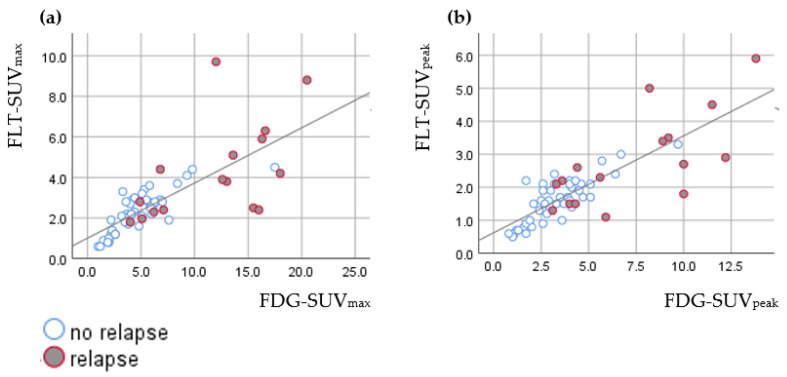
Correlation of (**a**) FDG-SUV_max_ and FLT-SUV_max_ with linear fit (R = 0.804; *p* < 0.001; spearman’s correlation), and (**b**) FDG-SUV_peak_ and FLT-SUV_peak_ with linear fit (R = 0.770; *p* < 0.001; spearman’s correlation).

SUV_max_ and SUV_peak_ in HDV were strongly correlated for both FDG and FLT (FDG-SUV: R = 0.958; *p* < 0.001; FLT-SUV: R = 0.958; *p* < 0.001). FDG-SUV and FLT-SUV in HDV had a positive and strong correlation (SUV_max_: R = 0.804; *p* < 0.001; SUV_peak_: R = 0.770; *p* < 0.001), as shown in Figure 3.

**Figure 4:**
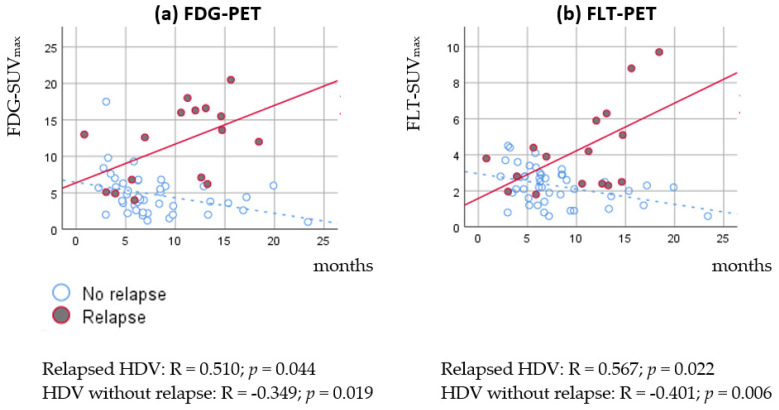
Scatter plot of SUV_max_ in HDV vs. time from the end of radiotherapy to the suspicion of relapse with a linear fit stratified by the relapse status. (**a**) FDG-SUV_max_; (**b**) FLT-SUV_max_.

FDG-SUV_max_ and FLT-SUV_max_ both decreased with time since radiotherapy in HDV without relapse (negative correlation, FDG-SUV_max_: R = −0.349; *p* = 0.019; FLT-SUV_max_: R = −0.401; *p* = 0.006), and increased with time in HDV with relapse (FDG-SUV_max_: R = 0.510; *p* = 0.044; FLT-SUV_max_: R = 0.567; *p* = 0.022). As illustrated in Figure 4, the difference of SUV_max_ in HDV with relapse vs. no-relapse increases with time from the end of radiotherapy.

Similar results were obtained when the analyses were restricted to include only patients with NSCLC (Supplemental Tables S2 and S3).

There was no significant correlation between the time from relapse suspicion to FDG-PET/CT and FDG-SUV_max_ in recurrent pulmonary lesions (*p* = 0.512), nor from the suspicion of relapse to FLT-PET/CT and FLT-SUV_max_ in recurrent lesions (*p* = 0.670), implying that SUV-estimates were not biased from the time delay between the relapse suspicion and conducting the PET scans.

### 3.2. Relapse Detection

**Figure 5:**
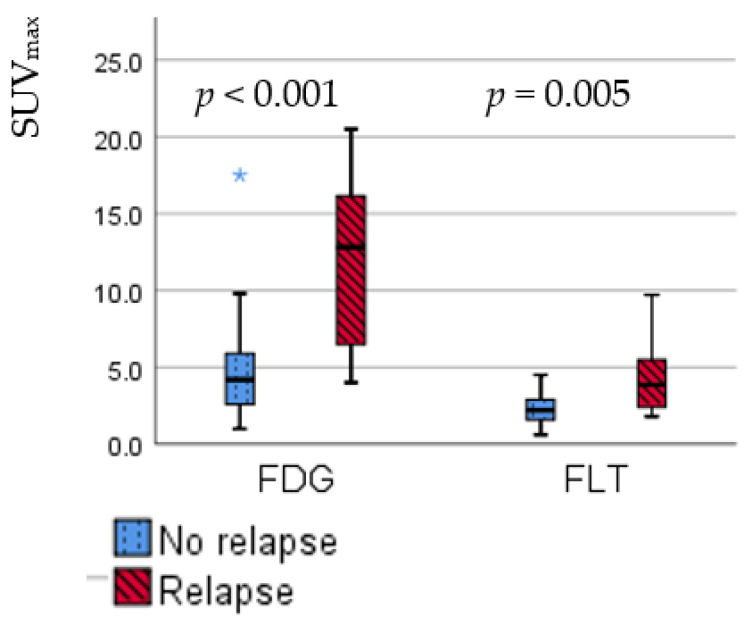
Boxplots of FDG- and FLT-SUV_max_ in HDV with relapse vs. HDV without relapse in patients with irradiated lung cancer.

The HDV with relapse had a significantly higher SUV_max_ compared with non-relapsed HDV (median FDG-SUV_max_: 12.8 vs. 4.2; *p* < 0.001; median FLT-SUV_max_ 3.9 vs. 2.2; *p* = 0.005), as illustrated in Figure 5. FDG-SUV_peak_ and FLT-SUV_peak_ showed similar results (median FDG-SUV_peak_: 7.1 vs. 3.2; *p* < 0.001; median FLT-SUV_peak_: 2.5 vs. 1.6; *p* = 0.005).

PET-parameters from pulmonary recurrent lesions are presented in Table 2. Recurrent lesions within HDV had higher SUV and larger functional tumor volumes than lesions with recurrence outside of HDV. However, only significantly so for FDG-PET-parameters (FDG-SUV_peak_, MTV_3.0_, and MTV_50%_).

#### 3.2.1. Diagnostic Accuracy of FDG-SUV_max_ and FLT-SUV_max_

**Figure 6:**
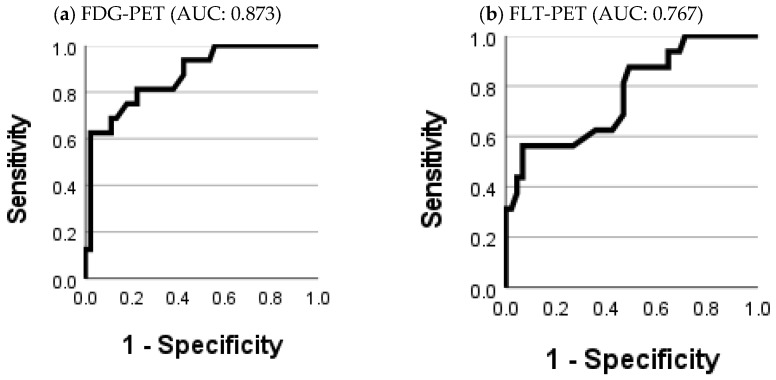
Receiver operating characteristic (ROC) curves for the relapse diagnosis with FDG-SUV_max_ (**a**) and FLT-SUV_max_ (**b**).

**Figure 7:**
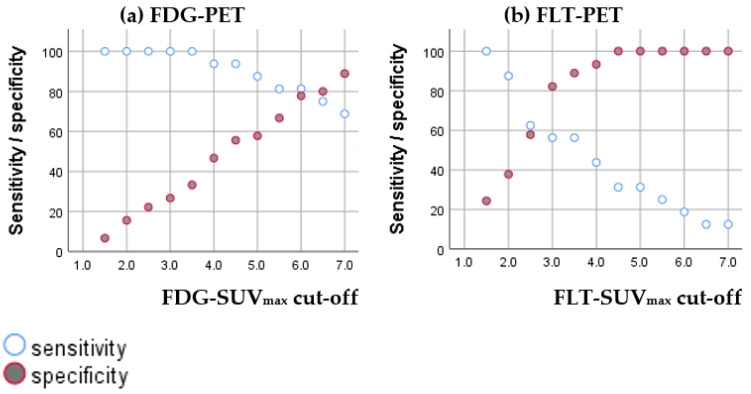
Scatter plot of sensitivity and specificity of FDG-SUV_max_ (**a**) and FLT-SUV_max_ (**b**) in HDV with variable cutoffs.

ROC-curves showed a moderate diagnostic accuracy for both FDG-SUV_max_ (AUC: 0.873) and FLT-SUV_max_ (AUC: 0.767), as shown in Figure 6. It was not possible from the ROC curves to establish cutoffs with a reasonable balance between sensitivity and specificity for neither FDG-SUV_max_ nor FLT-SUV_max_. The sensitivity and specificity for variant cutoffs of SUV_max_ are presented in Figure 7.

#### 3.2.2. Diagnostic Value of Combining FDG-SUV_max_ and FLT-SUV_max_

A superior diagnostic value of combining FDG-SUV_max_ and FLT-SUV_max_ was not obvious due to the high correlation of FDG-SUV_max_ and FLT-SUV_max_. Combinations of FDG-SUV_max_ and FLT-SUV_max_ with variant cutoffs were explored without an additional diagnostic value. In the subgroup of HDVs with high FDG-SUV_max_ (variant cutoffs), no optimal cutoff could be identified by the ROC-curves for FLT-SUV_max_ and the diagnostic accuracy was low (e.g., HDV with FDG-SUV_max_ ≥ 6.0: AUC: 0.654).

### 3.3. Prognosis

At the time of analysis, 33 patients had died. The median overall survival from relapse suspicion was 41 months. The median survival of patients who had intra-pulmonary relapse was 42 months. The two patients with extra-pulmonary relapse only died 7 and 10 months after relapse suspicion. The median overall survival of patients without relapse could not be estimated due to too few events (*n* = 12), and was at least 41 months.

In the univariate analysis, PET-parameters in HDV did not show a significant prognostic value of overall survival in the entire population (FDG-SUV_max_: *p* = 0.154; FLT-SUV_max_: *p* = 0.856), in patients with intrapulmonary relapse (*n* = 30; FDG-SUV_max_: *p* = 0.431; FLT-SUV_max_: *p* = 0.782), patients with HDV-relapse (*n* = 14; FDG-SUV_max_: *p* = 0.391; FLT-SUV_max_: *p* = 0.423), nor in patients without relapse (*n* = 29; FDG-SUV_max_: *p* = 0.314; FLT-SUV_max_: *p* = 0.862).

The univariate survival analysis performed on the patients with intrapulmonary relapse, appointed PTV_50%_ as the only significant prognostic factor for overall survival (HR: 1.07 pr cm^3^ [1.01–1.13]; *p* = 0.014). MTV_50%_, MTV_80%_, PTV_80%_, and sex were borderline significant. Results from the univariate analyses are shown in Table 3.

MTV_50%_ and MTV_80%_ were highly correlated (R = 0.785, *p* < 0.001), as were PTV_50%_ and PTV_80%_ (R = 0.531, *p* = 0.003). Therefore, only MTV_50%_ and PTV_50%_ were included in the multivariate analysis. Male sex was independently prognostic for a poorer overall survival. No PET-parameter had an independent prognostic value. Larger MTV_50%_ and a higher age were borderline significant for poorer overall survival. Results from the multivariate analysis are shown in Table 4. The subgroup analysis on patients with NSCLC yielded similar results (Supplemental Tables S4 and S5).

## 4. Discussion

We investigated the impact of semi-quantitative parameters of FDG-PET and FLT-PET in patients suspected for having a relapse of lung cancer after treatment with definitive radiotherapy. The FDG- and FLT-uptake was significantly higher in relapsed HDVs than HDVs without relapse. However, with a considerable overlap of SUVs in relapsed vs. non-relapsed HDVs, neither FDG-SUV_max_ nor FLT-SUV_max_ can be used definitively for distinguishing the relapse from radiation-induced changes. However, the longer after radiotherapy the PET scan was performed, the difference between SUV_max_ in HDVs with relapse vs. HDVs without relapse increased. The prognostic value of FDG- and FLT-PET-parameters after radiotherapy was limited.

Results from the few studies addressing PET-parameters in relapses after radiotherapy were similar to ours. Nakajima et al. [6] showed that patients with recurrences after SBRT had a significantly higher FDG-SUV_max_ than patients without relapse. Saga et al. [18] reported that after SBRT, FLT-SUV_max_ in relapsed lesions (range: 2.0–5.9; *n* = 5) were higher than in non-relapsed lesions (range: 1.3–2.1, *n* = 3; no statistics performed).

In our data, FDG- and FLT-uptakes in non-relapsed HDVs were lower the longer after radiotherapy PET/CT was performed, whereas FDG- and FLT-uptakes were higher in HDVs with relapse the longer after radiotherapy. PET-parameters may have a higher impact late after radiotherapy, as the difference of tracer-uptake in relapsed vs. non-relapsed lesions increased with time. In FDG-PET, a reduced FDG-uptake with time was expected following the resolution of radiation-induced changes [7,27]. FLT-PET is less likely to have an unspecific uptake in inflammation [28,29,30]. However, a moderate FLT-uptake the first 9 months after radiotherapy, as seen in our study, implies that some degree of non-specific FLT-uptake may be present.

It has previously been shown that the diagnostic accuracy of FDG-SUV_max_ was higher when FDG-PET/CT was performed more than 12 months after SBRT [6]. The sensitivity and specificity were 100% for SUV_max_ > 4.5 for patients who had FDG-PET/CT conducted more than 12 months after SBRT, compared with 87% and 98% for their entire cohort. Hoopes et al. [8] did not support a specificity of 100% of SUV_max_ > 4.5, as FDG-SUV_max_ was 5.1 and 5.9 in two patients without evidence of disease 23 and 26 months after SBRT, respectively [8]. A direct comparison of SUV_max_ from different studies should be taken with precaution. Particular precautions when comparing our study with the study of Nakajima et al. [6] are the different reconstruction methods (OSEM with and without PSF point-spread function, respectively) and different FDG-uptake times (60 vs. 90 min). PSF reconstructions lead on average to higher SUV_max_ than non-PSF reconstructions [31,32]. However, in our study, two patients without relapse had a high FDG-uptake on FDG-PET/CT more than 12 months after the end of radiotherapy: In one patient who had ended SBRT 14 months prior to FDG-PET/CT, FDG-SUV_max_ was 5.9. In another patient who had ended normo-fractionated radiotherapy 21 months prior to FDG-PET/CT, FDG-SUV_max_ was 6.0. FLT-SUV_max_ in the two patients were 2.5 and 2.2, respectively. In both patients, the biopsy was negative and there was no evidence of progression the following 6 months. Accordingly, from our data, the specificity of SUV_max_ > 4.5 was 75% in patients with relapse suspicion more than 12 months after the end of radiotherapy. The diagnostic value of the visual interpretation of FDG-PET/CT after treatment with a curative intent, surgery, and radiotherapy has been addressed in several papers with an estimated sensitivity between 82% and 100%, and specificity between 82% and 98% [2]. A meta-analysis showed a pooled sensitivity and specificity of 90% [33]. After radiotherapy, we have previously demonstrated a higher sensitivity and specificity with visual analysis of FDG-PET/CT (94%, and 71%, respectively) and FLT-PET/CT (69% and 90%, respectively) than any SUV_max_ cut-off [4]. From our data, we cannot support a fixed threshold of neither FDG-SUV_max_ nor FLT-SUV_max_ for the relapse diagnosis.

In this study, we also observed that relapses within HDV had higher SUV_max_, SUV_peak_, and larger tumor volumes than relapsed lesions that had not been irradiated previously, particularly for FDG-parameters. A non-malignant FDG-uptake caused by radiation-induced changes [6] could attribute to the differences, though also difficulties in the diagnostic process leading to later diagnosis of relapses within HDVs may cause larger and more aggressive tumor relapses in HDVs.

A limitation to our study was that the pathological confirmation of relapse was not always obtained. To minimize the effect of this limitation, a retrospective compound reference standard for the relapse status was applied, based on the patients’ records within 6 months of follow-up. In most patients, the relapse was confirmed by histology or follow-up with subsequent progression, and non-relapse by follow-up without progression. However, in 13/61 patients (six HDVs), the relapse was decided based on FDG-PET/CT by a multi-disciplinary conference, and the clinical decision warranted the start of treatment rather than further confirmation, either due to an obvious outcome of FDG-PET/CT and/or due to fact that the patient was unfit for invasive procedures. FDG-PET/CT is recommended as a second step test when the relapse is suspected after radiotherapy [3], thus excluding patients with an obvious relapse on FDG-PET/CT would have caused the study to be less representative.

Our survival analysis showed that FLT-PET (PTV_50%_) had a prognostic value in patients with relapsed lung cancer after radiotherapy. However, when adjusting for sex, age and time since RT, PTV_50%_ was not independently prognostic for overall survival. MTV_50%_ was borderline significant in both univariate and multivariate analysis. Therefore, MTV_50%_ might be a better prognosticator than PTV_50%_, but needs further confirmation.

Previous studies exploring the prognostic value of post-radiotherapy PET-parameters are limited, and most studies have focused on an FDG-change rather than the absolute value of PET-parameters [2]. In contrast to our results, Lopez Guerra et al. [20] showed a prognostic value of FDG-SUV_max_ after normo-fractionated radiotherapy for overall survival with HR 1.27. In addition, Bollineni et al. [21] showed a trend for the prognostic value of survival after SBRT [21]. In studies including lung cancer patients treated with surgery [2], and in studies including cohorts mixed of patients treated with a curative intent, the post-treatment FDG-SUV_max_ also showed a prognostic value [2,34]. FLT-SUV_max_ after the end of carbon-ion radiotherapy has not shown a prognostic value [19]. The above studies address different populations with different stages. However, they have in common that all patients have been treated with a curative intent. Baseline studies have previously shown a prognostic value of FDG-SUV_max_ and MTV [35,36]. Results from baseline FLT-PET studies are not in agreement [16,19,37], and Scheffler et al. [37] showed an independent prognostic value of FDG-SUV_max_, but not FLT-SUV_max_. The prognostic value of PET-parameters measured before the treatment may, however, not be applicable on PET-parameters measured after radiotherapy, as the more complex biological tumor characteristics may affect the tracer-uptake. In addition, the lesser treatment options for relapsed cancers affect the survival compared with survival in patients with treatment naïve tumors. The survival analysis in our study was limited by the sample size. Our cohort consisted of patients with different stages, different histologies, different treatment schemes, and accordingly a different prognosis, although all the patients were initially treated with a curative intent. A prognostic value of PET-parameters may be affected by other prognostic factors. However, the small patient number limited possibilities for adjustment. The overall survival in our study was long, also for patients with relapse. PET-parameters measured several years prior to death probably contain a less prognostic value. Approximately half of the patients with relapse were treated with a curative intent. However, the intent or relapse treatment did not have a prognostic value. Therefore, in this study, the overall survival may not be useful as an endpoint. The progression free survival was not selected as an endpoint, as patients were included due to a suspicion of relapse, and therefore, many patients were confirmed with relapse shortly after inclusion.

An early diagnosis of lung cancer relapse is essential, as in some cases, a curative intended treatment might be feasible [1]. In this study, relapsed lesions within HDV had higher FDG-SUV_peak_ and larger MTV, than the relapsed lesions outside of HDV. The same characteristics were applied for the FLT-uptake and PTV, though not statistically significant. The cause is unknown, however, it is possible that radiation-induced changes in the HDV challenge and thus delay the diagnostic decision making. FDG-PET-SUV_max_ and FLT-PET-SUV_max_ can be supportive for distinguishing between radiation-induced changes and relapse, but should not be used definitively. Over time, the differences in FDG-SUV_max_ and FLT-SUV_max_, respectively in relapsed vs. non-relapsed HDVs increased, thus SUV_max_ may have a higher impact later after radiotherapy.

## 5. Conclusions

The FDG- and FLT-uptake was significantly higher in relapsed HDV than HDV without relapse. However, caution should be taken if using SUV for the relapse diagnosis due to a considerable overlap of FDG-SUV_max_, respectively, FLT-SUV_max_ in relapsed vs. non-relapsed HDVs.

PTV_50%_ was prognostic for the overall survival in patients with relapse, however, PTV_50%_ had no independently prognostic value.

## Figures and Tables

**Table 1:** Patient characteristics, time logistics, and outcome (*n* = 61).

Patient Characteristics		Number/Median
Age, at day of suspicion (median (IQ))		70 (65–77)
Sex (male/female)		34/27
Histology	Adenocarcinoma	29
	Squamous cell carcinoma	25
	SCLC	2
	Not otherwise specified (NOS)	3
	Mixed NSCLC/SCLC	2
Stadium:	IA	12
	Ib	6
	IIa	2
	IIb	5
	IIIa	14
	IIIb	16
	IV	6
Chemotherapy (yes/no)		34/27
Radiotherapy:	SBRT (45-72 Gy)	28
	Normofractionated radiotherapy (66 Gy)	29
	Hyperfractionated radiotherapy (45-60 Gy)	3
	Normofractionated radiotherapy (66 Gy) and SBRT (45 Gy)	1
**Timing between scans**		
Time from the end of radiotherapy to relapse suspicion; in months (median (IQ))		7 (5–12)
Time from relapse suspicion to FDG-PET/CT; in days (median (IQ))		21 (15–27)
Time from relapse suspicion to FLT-PET/CT; in days (median (IQ))		23 (21–29)
Time between FLT-PET/CT and FDG-PET/CT; in days (median (IQ))		6 (3–9)
**Outcome**		
Overall relapse (yes/no)		32/29
HDV relapse (yes/no)		16/45
Intra-pulmonary relapse (yes/no)		30/31
Extra-pulmonary relapse (yes/no)		7/54
Deceased (yes/no)		33/28
Follow-up (from suspicion of relapse); in months (median (IQ))		25 (16–44)

IQ: Interquartile range; SCLC: Small cell lung cancer; NSCLC: Non-small cell lung cancer; SBRT: Stereotactic body radiotherapy; HDV: High-dose irradiated volume (>50% of prescribed dose).

**Table 2:** PET-parameters in intrapulmonary malignant lesions in patients with relapsed lung cancer.

FDG-PET-Parameters	All Lesion Median [IQ] (*n* = 30)	Lesions within HDV Median [IQ] (*n* = 16)	Lesions Outside of HDV Median [IQ] (*n* = 14)	*p*-Value
SUV_max_	8.6 [5.1–16.0]	12.8 [6.4–16.2]	6.2 [3.3–13.7]	0.095
SUV_peak_	4.7 [3.1–9.0]	7.1 [4.1–10.0]	3.5 [1.9–5.7]	0.014 *
MTV_3.0_	1.7 [0.5–10.5]	6.6 [1.6–52.2]	0.9 [0.2–2.0]	0.016 *
MTV_80%_	0.1 [0.1–0.5]	0.2 [0.1–0.6]	0.1 [0.1–0.2]	0.113
MTV_50%_	1.2 [0.5–3.6]	2.5 [1.2–7.9]	0.7 [0.4–1.2]	0.014 *
**FLT-PET-parameters**				
SUV_max_	3.7 [2.0–5.1]	3.9 [1.2–7.9]	3.3 [1.4–4.4]	0.188
SUV_peak_	2.3 [1.3–3.3]	2.5 [1.6–3.5]	2.0 [1.0–2.9]	0.145
PTV_3.0_	0.2 [0.0–2.0]	0.3 [0.0–4.0]	0.1 [0.0–0.4]	0.128
PTV_80%_	0.2 [0.1–0.3]	0.3 [0.1–0.5]	0.1 [0.1–0.2]	0.311
PTV_50%_	1.3 [0.8–4.3]	2.7 [0.8–10.1]	1.2 [0.5–1.5]	0.066

MTV: Metabolic tumor volume; PTV: Proliferative tumor volume. * Statistically significant.

**Table 3:** Prognostic value of PET-parameters and clinical variables in patients with relapsed lung cancer (*n* = 30). Univariate survival analysis.

FDG-PET-Parameters in Recurrent Lesions, Continuous Variable	Hazard Ratio [95% CI]	*p*-Value
SUV_max_ (per unit)	1.02 [0.94–1.10]	0.675
SUV_peak_ (per unit)	1.07 [0.94–1.21]	0.335
MTV_3.0_ (per cm^3^)	1.01 [0.99–1.02]	0.485
MTV_80%_ (per cm^3^)	3.63 [0.95–13.91]	0.060
MTV_50%_ (per cm^3^)	1.13 [1.00–1.27]	0.054
**FDG-PET-parameters in recurrent lesions, dichotomized variable**		
SUV_max_ (>8.6)	1.09 [0.43–2.76]	0.853
SUV_peak_ (>4.7)	1.17 [0.46–2.96]	0.741
MTV_3.0_ (>1.7 cm^3^)	0.81 [0.32–2.07]	0.665
MTV_80%_ (>0.1 cm^3^)	1.43 [0.56–3.64]	0.454
MTV_50%_ (>1.2 cm^3^)	1.56 [0.61–3.97]	0.355
**FLT-PET-parameters in recurrent lesions, continuous variable**		
SUV_max_ (per unit)	0.93 [0.75–1.15]	0.496
SUV_peak_ (per unit)	0.92 [0.64–1.31]	0.635
PTV_3.0_ (per cm^3^)	1.05 [0.94–1.17]	0.411
PTV_80%_ (per cm^3^)	4.13 [0.85–20.16]	0.080
PTV_50%_ (per cm^3^)	1.07 [1.01–1.13]	0.014 *
**FLT-PET-parameters in recurrent lesions, dichotomized variable**		
SUV_max_ (>3.7)	0.80 [0.31–2.01]	0.644
SUV_peak_ (>2.3)	0.96 [0.38–2.47]	0.939
PTV_3.0_ (>0.2 cm^3^)	0.78 [0.30–2.07]	0.619
PTV_80%_ (>0.2 cm^3^)	1.67 [0.65–4.26]	0.285
PTV_50%_ (>1.3 cm^3^)	1.42 [0.56–3.61]	0.465
**Clinical parameters**		
Age (at suspicion) (per year)	1.04 [0.97–1.11]	0.268
Sex (male)	2.74 [0.98–7.67]	0.055
Stadium (III vs. I–II)	1.18 [0.45–3.14]	0.736
(IV vs. I–II)	0.59 [0.07–4.83]	0.622
Radiotherapy (Conventionally fractionated radiotherapy vs. SBRT)	0.97 [0.38–2.52]	0.956
Histology (Squamous cell carcinoma vs. adenocarcinoma)	1.24 [0.48–3.20]	0.659
Time since the end of radiotherapy (per month)	0.99 [0.90–1.09]	0.837
Site of relapse (within HDV vs. outside HDV)	1.53 [0.59–3.94]	0.381
Intention for the relapse treatment (palliation vs. curative)	1.33 [0.51–3.46]	0.564
Extra-pulmonary metastases (present)	2.18 [0.70–6.77]	0.180

* Statistically significant.

**Table 4:** Multivariate survival analysis in patients with relapsed lung cancer.

Covariate	Hazard Ratio [95% CI]	*p*-Value
MTV_50%_ (per cm^3^)	1.19 [0.99–1.44]	0.063
PTV_50%_ (per cm^3^)	1.02 [0.95–1.09]	0.650
Sex (male)	4.32 [1.34–13.92]	0.014 *
Age (at suspicion) (per year)	1.08 [1.00–1.17]	0.054
Time since the end of radiotherapy (per month)	0.96 [0.88–1.05]	0.349

* Statistically significant.

## Data Availability

The data presented in this study are available on reasonable request from the corresponding author.

## Supplementary Materials

The following are available online at https://www.mdpi.com/2075-4418/11/2/279/s1. Table S1: Data on the PET model and reconstruction used for FDG-PET/CT; Table S2: PET-parameters in intrapulmonary malignant lesions in patients with NSCLC; Table S3: FDG-SUV_max_ and FLT-SUV_max_ in relapsed vs. non-relapsed HDV in patients with NSCLC; Table S4: Prognostic value of PET-parameters and clinical variables in patients with intrapulmonary relapse of irradiated NSCLC; Table S5: multivariate survival analysis in patients with relapsed NSCLC.
