# Correction: Christensen et al. Impact of [^18^F]FDG-PET and [^18^F]FLT-PET-Parameters in Patients with Suspected Relapse of Irradiated Lung Cancer. *Diagnostics* 2021, *11*, 279

**DOI:** 10.3390/diagnostics12071741

**Published:** 2022-07-18

**Authors:** Tine N. Christensen, Seppo W. Langer, Gitte Persson, Klaus Richter Larsen, Annemarie G. Amtoft, Sune H. Keller, Andreas Kjaer, Barbara Malene Fischer

**Affiliations:** 1 Department of Clinical Physiology, Nuclear Medicine & PET, Copenhagen University Hospital, Rigshospitalet, 2100 Copenhagen Ø, Denmark; annemarie.gjelstrup.amtoft@regionh.dk (A.G.A.); sune.hoegild.keller@regionh.dk (S.H.K.); andreas.kjaer@regionh.dk (A.K.); barbara.malene.fischer@regionh.dk (B.M.F.); 2 Cluster for Molecular Imaging, University of Copenhagen, 2200 Copenhagen N, Denmark; 3 Department of Oncology, Copenhagen University Hospital, Rigshospitalet, 2100 Copenhagen Ø, Denmark; seppo.Langer@regionh.dk; 4 Department of Clinical Medicine, University of Copenhagen, 2100 Copenhagen Ø, Denmark; gitte.persson@regionh.dk; 5 Department of Oncology, Herlev-Gentofte Hospital, University of Copenhagen, 2730 Herlev, Denmark; 6 Department of Pulmonary Medicine, Bispebjerg University Hospital, 2400 Copenhagen NV, Denmark; klaus.richter.larsen@regionh.dk; 7 The PET Centre, School of Biomedical Engineering and Imaging Science, King’s College London, London SE1 7EH, UK

## Error in Figure

In the original publication [1], there was a mistake in Figure 6a. Figure 6a was replaced by Figure 6b, and thus Figure 6a,b appeared identical. The corrected Figure 6 appears below.

The authors apologize for any inconvenience caused and state that the scientific conclusions are unaffected. This correction was approved by the Academic Editor. The original publication has also been updated.

## Figures and Tables

**Figure 6:**
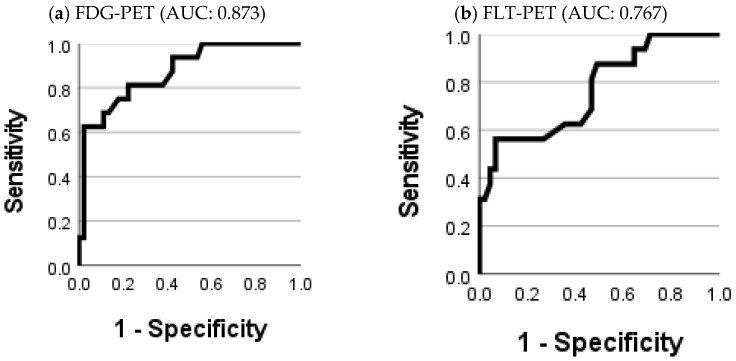
Receiver operating characteristic (ROC) curves for the relapse diagnosis with FDG-SUV_max_ (**a**) and FLT-SUV_max_ (**b**).

## References

[1] Christensen T.N., Langer S.W., Persson G., Larsen K.R., Amtoft A.G., Keller S.H., Kjaer A., Fischer B.M. (2021). Impact of [^18^F]FDG-PET and [^18^F]FLT-PET-Parameters in Patients with Suspected Relapse of Irradiated Lung Cancer. Diagnostics.

