# Supplementary material for: Impact of [18F]FDG-PET and [18F]FLT-PET-Parameters in Patients with Suspected Relapse of Irradiated Lung Cancer
Source: Diagnostics (Basel). 2021 Feb 11;11(2):279. doi: 10.3390/diagnostics11020279 (PMC7916960; doi:10.3390/diagnostics11020279)
Supplement: Supplementary file 1 [file diagnostics-11-00279-s001.zip › diagnostics-1065899-supplementary.pdf]

Supplemental Table S1 Data on PET model and reconstruction used for FDG-PET/CT

Hospital	PET manufacturer	PET model	Reconstruction method	Beta, or iterations and subsets	Postreconstruction filter (FWHM)	No of patients
Bispebjerg Hospital	GE Medical Systems	Discovery 710	QCFX	β=500	-	4
		Discovery MI	QCFX	β=500	-	1
	Philips Medical Systems	GEMINI TF 64	BLOB-OS-TF	3i33s	λ=1	4
Herlev Hospital	Siemens	Biograph mCT 64	OSEM PSF+TOF	4i21s	2.5 mm	1
Rigshospitalet	Siemens	Biograph mCT 64	OSEM PSF+TOF	2i21s	2.0 mm	20
			OSEM PSF+TOF	2i21s	2.5 mm	1
		Biograph TruePoint TrueV 40	OSEM PSF	3i21s	2 mm	14
			OSEM PSF	3i21s	2.5 mm	1
		Biograph TruePoint TrueV 64	OSEM PSF	3i21s	2.0 mm	14
			OSEM PSF	3i21s	2.5 mm	1

FDG-PET/CT: 2-deoxy-2-[^{18}F]fluoro-D-glucose-PET/CT; QCFX: QClear with PFS and filtering; BLOB-OS-TF: OSEM with blobs and TOF; OSEM: Ordered subset expectation maximization; PSF: point spread function; TOF: time of flight; I: iterations; S: subsets

Supplemental Table S2 PET-parameters in intrapulmonary malignant lesions in patients with NSCLC

**FDG-PET-parameters**	**All lesions median [IQ] (*n* = 28)**	**Within HDV median [IQ] (*n* = 15)**	**Outside of HDV median [IQ] (*n* = 13)**	***p*-value**
SUV$_{max}$	10.6 [5.1-16.1]	13.0 [6.8-16.3]	7.2 [4.1-14.5]	0.106
SUV$_{peak}$	5.0 [3.2-9.1]	8.2 [4.3-10.0]	3.9 [2.3-6.2]	0.017*
MTV$_{3.0}$	1.8 [0.6-11.8]	6.6 [1.6-52.2]	1.2 [0.2-2.2]	0.017*
MTV$_{80\%}$	0.1 [0.1-0.5]	0.2 [0.1-0.6]	0.1 [0.1-0.1]	0.112
MTV$_{50\%}$	1.2 [0.4-3.9]	2.5 [1.2-7.9]	0.6 [0.4-1.2]	0.014*
**FLT-PET-parameters**				
SUV$_{max}$	3.8 [2.0-5.1]	3.9 [2.4-5.9]	3.6 [1.5-3.1]	0.235
SUV$_{peak}$	2.4 [1.4-3.4]	2.6 [1.5-3.5]	2.2 [1.1-3.1]	0.194
PTV$_{3.0}$	0.2 [0.0-2.0]	0.3 [0.0-4.5]	0.1 [0.0-0.5]	0.128
PTV$_{80\%}$	0.2 [0.1-0.3]	0.3 [0.1-0.5]	0.1 [0.1-0.2]	0.406
PTV$_{50\%}$	1.3 [0.8-4.3]	2.7 [0.8-10.1]	1.2 [0.6-1.7]	0.072

NSCLC: non-small cell lung cancer; HDV: high-dose irradiated volume (>50% of prescribed dose); IQ: interquartile range; SUV: standardized uptake value; MTV: metabolic tumour volume; PTV: proliferative tumour volume; * Statistically significant.

Supplemental Table S3 FDG-SUV_{max} and FLT-SUV_{max} in relapsed vs. non-relapsed HDV in patients with NSCLC

	**HDV with relapse Mean (*n* = 15)**	**HDV without relapse Mean (*n* = 42)**	**Mean difference**	***p*-value**
FDG-SUV_{max}	12.0	4.8	7.2 [4.1-10.4]	0.0001*
FLT-SUV_{max}	4.4	2.3	2.1 [0.7-3.5]	0.006*

SUV: standardized uptake value; HDV: high-dose irradiated volume (>50% of prescribed dose); NSCLC: non-small cell lung cancer; * Statistically significant.

Supplemental Table S4 Prognostic value of PET-parameters and clinical variables in patients with intrapulmonary relapse of irradiated NSCLC. Univariate survival analysis (*n* = 28)

**FDG-PET parameters in recurrent lesions, continuous variable**	**Hazard ratio [95% CI]**	***p*-value**
SUV_{max} (pr unit)	1.00 [0.93-1.08]	0.962
SUV_{peak} (pr unit)	1.05 [0.92-1.19]	0.508
$MTV_{3.0}$ (pr cm^3)	1.01 [0.99-1.02]	0.560
$MTV_{80\%}$ (pr cm^3)	3.51 [0.93-13.20]	0.064
$MTV_{50\%}$ (pr cm^3)	1.12 [0.99-1.26]	0.067
**FDG-PET parameters in recurrent lesions, dichotomized variable**		
SUV_{max} (> 10.6)	1.01 [0.40-2.56]	0.979
SUV_{peak} (> 4.9)	1.01 [0.40-2.56]	0.979
$MTV_{3.0}$ (> 1.7 cm^3)	0.75 [0.29-1.91]	0.547
$MTV_{80\%}$ (> 0.1 cm^3)	1.58 [0.62-4.01]	0.340
$MTV_{50\%}$ (> 1.2 cm^3)	1.45 [0.57-3.70]	0.436
**FLT-PET parameters in recurrent lesions, continuous variable**		
SUV_{max} (pr unit)	0.90 [0.73-1.12]	0.340
SUV_{peak} (pr unit)	0.88 [0.62-1.26]	0.489
$PTV_{3.0}$ (pr cm^3)	1.04 [0.92-1.17]	0.519
$PTV_{80\%}$ (pr cm^3)	3.72 [0.73-18.87]	0.113
$PTV_{50\%}$ (pr cm^3)	1.07 [1.01-1.13]	0.018*
**FLT-PET parameters in recurrent lesions, dichotomized variable**		
SUV_{max} (> 3.7)	0.69 [0.27-1.76]	0.435
SUV_{peak} (> 2.4)	0.82 [0.32-2.10]	0.684
$PTV_{3.0}$ (> 0.2 cm^3)	0.68 [0.26-1.80]	0.438
$PTV_{80\%}$ (> 0.2 cm^3)	1.57 [0.62-4.02]	0.350
$PTV_{50\%}$ (> 1.3 cm^3)	1.31 [0.51-3.35]	0.570
**Clinical parameters**		
Age (at suspicion) (pr year)	1.03 [0.96-1.10]	0.453
Sex (male)	2.80 [1.00-7.84]	0.050
Stadium (III vs I-II)	1.15 [0.43-3.05]	0.779
(IV vs I-II)	0.54 [0.07-4.43]	0.567
Radiotherapy (Conventionally fractionated radiotherapy vs. SBRT)	1.28 [0.48-3.42]	0.625
Histology (Squamous cell carcinoma vs. adenocarcinoma)	1.24 [0.48-3.20]	0.659

Time since end of radiotherapy (pr month)	0.98 [0.90-1.08]	0.726
Site of relapse (within HDV vs. outside HDV)	1.54. [0.60-3.98]	0.373
Intention for relapse treatment (palliation vs. curative)	1.30 [0.50-3.39]	0.593
Extra-pulmonary metastases (present)	1.96 [0.63-6.11]	0.245

SUV: standardized uptake value; MTV: metabolic tumour volume; PTV: proliferative tumour volume; SBRT: stereotactic body radiotherapy; HDV: high-dose irradiated volume (>50% of prescribed dose); * Statistically significant.

Supplemental Table S5 Multivariate survival analysis in patients with relapsed NSCLC (*n* = 28)

**Covariate**	**Hazard ratio [95% CI]**	***p*-value**
$MTV_{50\%}$ (pr cm^3)	1.19 [0.98-1.44]	0.072
$PTV_{50\%}$ (pr cm^3)	1.02 [0.95-1.09]	0.638
Sex (male)	4.17 [1.28-13.56]	0.018*
Age (at suspicion) (pr year)	1.08 [1.00-1.17]	0.065
Time since end of radiotherapy (pr month)	0.96 [0.88-1.05]	0.355

MTV: metabolic tumour volume; PTV: proliferative tumour volume; * Statistically significant.
